# Trends in knee arthroscopy and subsequent arthroplasty in an Australian population: a retrospective cohort study

**DOI:** 10.1186/1471-2474-14-143

**Published:** 2013-04-23

**Authors:** Ian A Harris, Navdeep S Madan, Justine M Naylor, Shanley Chong, Rajat Mittal, Bin B Jalaludin

**Affiliations:** 1 South Western Sydney Clinical School, University of New South Wales, Liverpool, NSW, Australia; 2 Orthopaedic Department, Liverpool Hospital, Liverpool, NSW, Australia; 3 Centre for Research, Evidence Management and Surveillance, Liverpool, NSW, Australia

**Keywords:** Knee arthroscopy, Knee arthroplasty

## Abstract

**Background:**

Knee arthroscopy is a common procedure in orthopaedic surgery. In recent times the efficacy of this procedure has been questioned with a number of randomized controlled trials demonstrating a lack of effect in the treatment of osteoarthritis. Consequently, a number of trend studies have been conducted, exploring rates of knee arthroscopy and subsequent conversion to Total Knee Arthroplasty (TKA) with varying results. Progression to TKA is seen as an indicator of lack of effect of primary knee arthroscopy.

The aim of this paper is to measure overall rates of knee arthroscopy and the proportion of these patients that undergo subsequent total knee arthroplasty (TKA) within 24 months, and to measure trends over time in an Australian population.

**Methods:**

We conducted a retrospective cohort study of all adults undergoing a knee arthroscopy and TKA in all hospitals in New South Wales (NSW), Australia between 2000 and 2008. Datasets obtained from the Centre for Health Record Linkage (CHeReL) were analysed using negative binomial regression. Admission rates for knee arthroscopy were determined by year, age, gender and hospital status (public versus private) and readmission for TKA within 24 months was calculated.

**Results:**

There was no significant change in the overall rate of knee arthroscopy between 2000 and 2008 (-0.68%, 95% CI: -2.80 to 1.49). The rates declined in public hospitals (-1.25%, 95% CI: -2.39 to -0.10) and remained relatively steady in private hospitals (0.42%, 95% CI: -1.43 to 0.60). The proportion of patients 65 years or over undergoing TKA within 24 months of knee arthroscopy was 21.5%. After adjusting for age and gender, there was a significant decline in rates of TKA within 24 months of knee arthroscopy for all patients (-1.70%, 95% CI:-3.13 to -0.24), patients admitted to private hospitals (-2.65%, 95% CI: -4.06 to -1.23) and patients aged ≥65 years (-3.12%, 95% CI: -5.02 to -1.18).

**Conclusions:**

Rates of knee arthroscopy are not increasing, and the proportion of patients requiring a TKA within 24 months of a knee replacement is decreasing in the age group most likely to have degenerative changes in the knee.

## Background

Arthroscopy is an important procedure in orthopaedic surgery, having both diagnostic and therapeutic applications. Knee arthroscopy has become the gold standard in the diagnosis of meniscal and ligamentous injury [1], and also plays a role in their management [2]. In recent times, the use of knee arthroscopy for the management of osteoarthritis has also become widespread subsequent to studies demonstrating benefit [3-6]. However, most of these studies were case series or observational in nature and thus do not provide strong evidence [7]. Other studies have questioned the use of knee arthroscopy for degenerative knee disease [8]. Nonetheless, some predictors of successful outcome emerged [9]; younger patients [10] reporting predominantly mechanical symptoms [11] with normally aligned knees [12] and short duration of symptoms [12] were more likely to benefit from arthroscopy.

Recently, three randomised controlled trials [13-15] provided high level evidence that knee arthroscopy is ineffective in the management of symptomatic osteoarthritis, including those with mechanical symptoms and meniscus tears, compared to placebo or alternative (non-operative) treatment. In light of the disparate effectiveness of arthroscopy for different underlying conditions and age groups, a number of studies have been conducted exploring rates of knee arthroscopy for different age groups and indications [16-20]. Total Knee Arthroplasty (TKA) within 1–2 years following arthroscopy is viewed as an indicator of lack of effect of the arthroscopic procedure. Studies in other populations have reported conversion rates to TKA within two years following the procedure [18,20].

This study was undertaken to describe the age-standardised rates for knee arthroscopy in an Australian population together with the conversion rate to TKA. As the utilisation rates of knee arthroscopy have also been shown to differ according to socioeconomic status [17], and as access varies between public and private hospitals, we also report the utilisation rates according to hospital status (private versus public).

The utilisation rates of knee arthroscopy and the rate of conversion to TKA subsequent to knee arthroscopy have not yet been described for an Australian cohort, thus, the data will serve as a useful point of reference, and will allow comparison with international rates.

## Methods

Data from the New South Wales (NSW) Admitted Patient Data Collection (APDC) pertaining to arthroscopic surgery and TKA between 2000 and 2008 were obtained from the Centre for Health Record Linkage (CHeReL). The APDC contains mandatory data from all hospitals (public and private) and day-procedure centres in NSW. The Australian Classification of Health Intervention procedure codes were used to identify participants undergoing knee arthroscopy or TKA (Table 1). All arthroscopic codes were used except those for ligament reconstruction. Age-specific population estimates at 31 December of each of the years were derived from the Australian Bureau of Statistics, as these dates correspond to the mid-point of each financial year of hospitalisation.

**Table 1:** Procedure codes used for data extraction

49557-00	Arthroscopy of knee
49560-00	Arthroscopic removal of loose body of knee
49560-02	Arthroscopic lateral release of knee
49557-01	Arthroscopic biopsy of knee
49558-00	Arthroscopic debridement of knee
49560-01	Arthroscopic trimming of ligament of knee
49566-00	Arthroscopic synovectomy of knee
49557-02	Arthroscopic excision of meniscal margin or plica of knee
49560-03	Arthroscopic meniscectomy of knee
49561-00	Arthroscopic lateral release of knee with debridement osteoplasty/chondroplasty
49561-01	Arthroscopic meniscectomy of knee with debridement, osteoplasty/chondroplasty
49561-02	Arthroscopic removal of loose body with debridement, osteoplasty/chondroplasty
49562-00	Arthroscopic lateral release with chondroplasty and multiple drilling or implant
49562-01	Arthroscopic meniscectomy with chondroplasty and multiple drilling or implant
49562-02	Arthroscopic removal of loose body, chondroplasty and multiple drilling/implant
49563-00	Arthroscopic repair of meniscus of knee
49558-01	Arthroscopic chondroplasty of knee
49558-02	Arthroscopic osteoplasty of knee
49559-00	Arthroscopic chondroplasty of knee with multiple drilling or implant
4951700	Hemiarthrosplasty of knee
4951800	Total arthrosplasty of knee, unilateral
4951900	Total arthrosplasty of knee, bilateral
4953401	Total Replacement arthroplasty of patellofemoral joint of knee
4952100	Total arthroplasty of knee with bone graft to femur, unilateral
4952101	Total arthroplasty of knee with bone graft to femur, bilateral
4952102	Total arthroplasty of knee with bone graft to tibia, unilateral
4952103	Total arthroplasty of knee with bone graft to tibia, bilateral
4952400	Total arthroplasty of knee with bone graft to femur and tibia, unilateral
4952401	Total arthroplasty of knee with bone graft to femur and tibia, bilateral
4953000	Revision of total arthroplasty of knee with bone graft to femur
4953001	Revision of total arthroplasty of knee with bone graft to tibia
4953300	Revision of total arthroplasty of knee with bone graft to femur and tibia
4955400	Revision of total arthroplasty of knee with anatomic specific allograft
4952700	Revision of total arthroplasty of knee

Following data custodian and NSW Population and Health Services Research Ethics Committee approvals, the CHeReL supplied lists of de-identified specific records.

Data in the NSW Admitted Patient Data Collection prior to 1 July 2000 were incomplete, and thus the timeframe of 1st July 2000 to 31st December 2008 (the most recent available data) was chosen for this study. Data retrieved included number of knee arthroscopies and TKA by age, gender, year and hospital status (private vs. public). Data on the indications for knee arthroscopy were found to be incomplete and therefore too unreliable to report. A routine quality check by CHeReL using a 1000-person sample from our data found a false link error rate of 4/1,000 records (0.4%).

Knee arthroscopy rates were calculated per 100 000 of population aged ≥18 years. The rate of TKA within 24 months of knee arthroscopy was generated by calculating the total number of TKA within 24 months of the date of initial knee arthroscopy, divided by total number of knee arthroscopies performed for each year. Negative binomial regression analyses were used to determine the percentage change in rate [21]. The main explanatory variable was year, with age and gender included as covariates.

Negative binomial regression models were applied to determine the percentage change in rate and to accommodate for over-dispersion in the data, with the log of NSW population used as an offset to control for the population changes over time.

## Results

### Knee arthroscopy rates by year and hospital status

**Figure 1:**
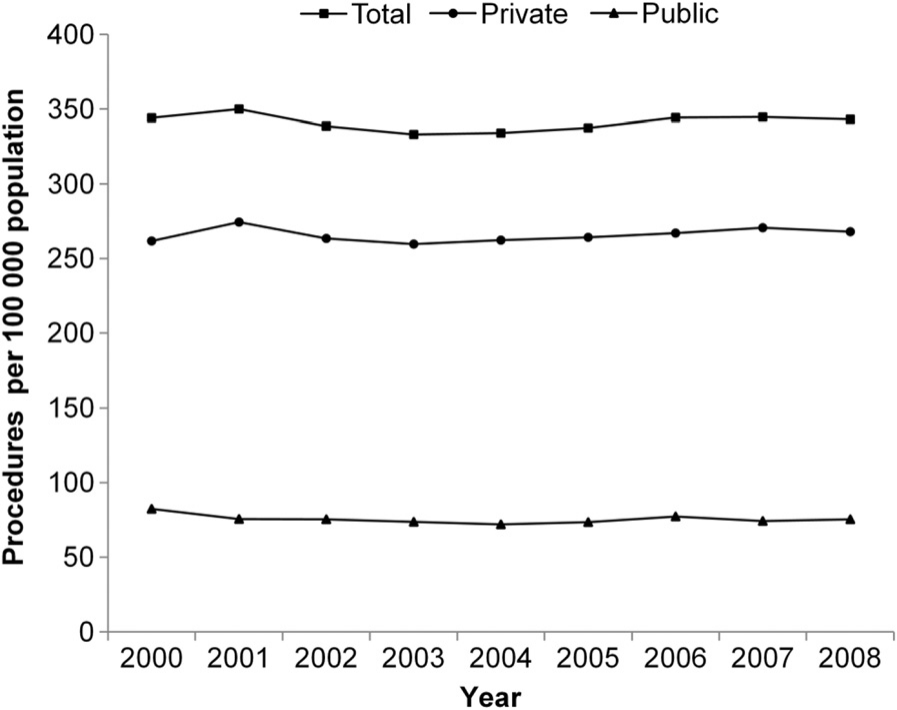
Total, public and private knee arthroscopy rates per 100 000 population between 2000 and 2008.

Descriptive statistics for all patients undergoing knee arthroscopy are provided in Table 2. The rate of knee arthroscopy was unchanged over the period studied: from 344.1 per 100,000 people in 2000 to 343.3 per 100,000 people in 2008 (change in rate: -0.68%, 95% CI: -2.80 – 1.49) (Figure 1). In public hospitals, there was a significant decline from 82.3 per 100 000 people in 2000 to 75.3 per 100 000 people in 2008 (change in rate: -1.25%, 95% CI: -2.39 − −0.10); in private hospitals, the rate changed from 261.8 per 100 000 people in 2000 to 268.0 per 100 000 people in 2008 (change in rate: -0.42%, 95% CI: -1.43 – 0.60). The ratio of the rate of private knee arthroscopies to public knee arthroscopies in NSW in 2008 was 3.6:1 compared to 3.2:1 in 2000. The total number of knee arthroscopies (not adjusted for population change) increased by 1.1% (p = 0.007) in public hospitals and by 13.0% (p < 0.001) in private hospitals between 2000 and 2008.

**Table 2:** Descriptive statistics for all arthroscopy patients, NSW 2000-2008

		**Number of patients (%)**
**Gender**	Male	94134 (59.5%)
	Female	64046 (40.5%)
**Age group (years)**	18-24	12337 (7.8%)
	25-34	20916 (13.2%)
	35-44	28988 (18.3%)
	45-54	36216 (22.9%)
	55-64	33221 (21.0%)
	65-74	18380 (11.6%)
	≥75	8122 (5.1%)
**Hospital type**	Public	34929 (22.1%)
	Private	123251 (77.9%)

### Knee arthroscopy rates by age group

**Figure 2:**
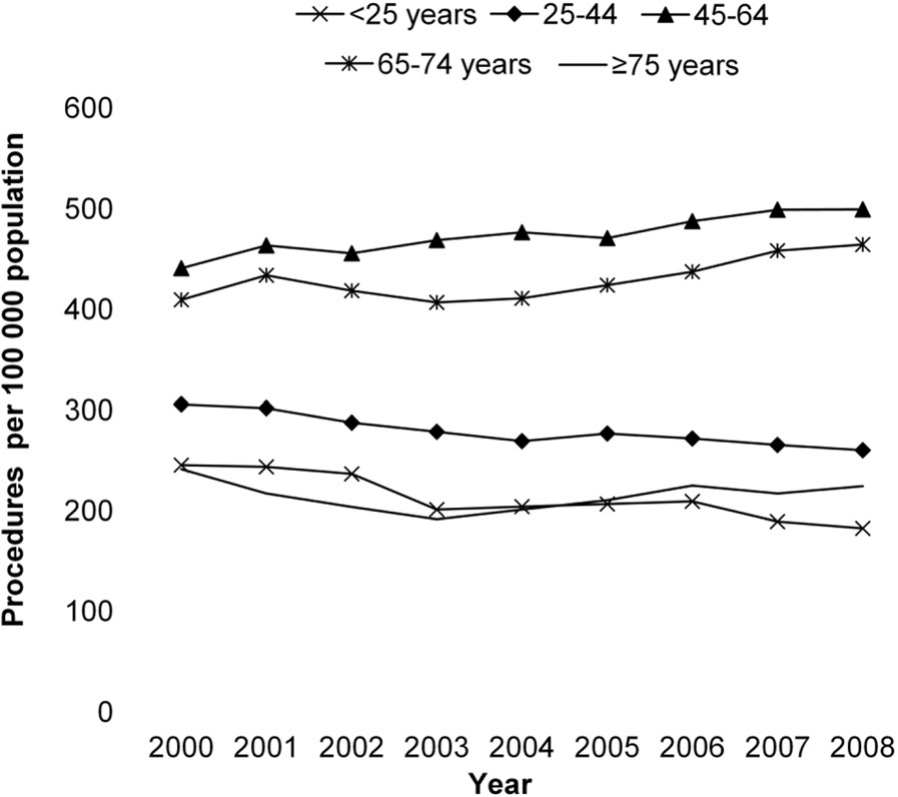
Rates of knee arthroscopy per 100 000 population between 2000 and 2008 by age group.

The rate of knee arthroscopy was also examined by age across the study period (Figure 2). After adjusting for sex, there was no significant change in rates of knee arthroscopy over time by age group (Table 3).

**Table 3:** Changes in knee arthroscopy rate between 2000 and 2008 for each age group

**Age group (years)**	**<25**	**25-34**	**35-44**	**45-54**	**55-64**	**65-74**	**≥75**
**Rate change**	−4.12%	−2.96%	−0.71%	0.94%	1.89%	1.01%	−1.02%
**95% CI**	−9.08–1.11	−7.96–2.31	−6.28– 5.17	−5.10–7.37	−3.69–7.78	−2.52–4.65	−5.89–4.09

### Rates of readmission for primary TKA within 24 months of knee arthroscopy

The rates of primary TKA within 24 months following knee arthroscopy for years 2000 to 2006 are shown in Table 4. After adjusting for age and sex, there was a significant decline in the overall rate of readmission for TKA (change in rate: -1.70%, 95% CI: -3.13 − −0.24), readmission rates in private hospitals (change in rate: -2.65%, 95% CI: -4.06 − −1.23) and in patients aged ≥65 years (change in rate: -3.12%, 95% CI: -5.02 − −1.18). In public patients the rates of conversion remained relatively steady (change in rate: -0.26%, 95% CI: -2.33 – 1.85). The rate of conversion to TKA was not calculated after 2006 as 24 month follow-up data was not available after that time.

**Table 4:** Rate of readmission for primary TKA within 24 months of knee arthroscopy

		**2000**	**2001**	**2002**	**2003**	**2004**	**2005**	**2006**	**Total**
**All ages**	TKA within 24 m of arthroscopy	1244	1361	1351	1313	1234	1246	1361	9110
	Total arthroscopies	16942	17469	17083	16947	17141	17478	18055	121115
	Proportion of total cases	7.3%	7.8%	7.9%	7.8%	7.2%	7.1%	7.5%	7.5%
**All ages (Private)**	TKA within 24 m of arthroscopy	966	1050	1013	1014	942	923	1030	6938
	Total arthroscopies	12892	13703	13287	13208	13457	13681	14006	94234
	Proportion of total cases	7.5%	7.7%	7.6%	7.7%	7.0%	6.8%	7.4%	7.4%
**All ages (Public)**	TKA within 24 m of arthroscopy	278	311	338	299	292	323	331	2172
	Total arthroscopies	4050	3766	3796	3739	3684	3797	4049	26881
	Proportion of total cases	6.9%	8.3%	8.9%	8.0%	7.9%	8.5%	8.2%	8.1%
**≥65 years**	TKA within 24 m of arthroscopy	656	689	614	604	564	551	614	4292
	Total arthroscopies	2832	2891	2790	2707	2799	2904	3049	19972
	Proportion of total cases	23.2%	23.8%	22.0%	22.3%	20.2%	19.0%	20.1%	21.5%

## Discussion

The rate of knee arthroscopy in NSW (334.0 per 100 000 in 2004) is higher than rates reported for England (<150 per 100 000 population in 2004) and also higher than those found in Canada (<200 per 100 000 population in 2004) [17]. However, the rate is lower than has been reported in the USA (404 per 100 000 population over a similar period) [22].

Over the 7.5-year period studied, there was a significant decline in the rates of knee arthroscopy in public hospitals whilst the rate remained relatively unchanged in private hospitals, and overall, the rate was higher in private hospitals. This difference may be due to a lack of access to the public sector, forcing uninsured patients into the private sector. Alternatively, differing thresholds for surgery may exist between private and public patients. Financial reimbursement has also been shown to influence surgical decision-making and the greater rates of knee arthroscopy in private hospitals may be related to greater financial incentives for surgeons [23]. The steady rates of knee arthroscopy in private hospitals may be explained by the increasing rate of private health insurance which has occurred in recent times; the rate of private insurance increased 2% (from 52% to 54%) for NSW between 2000 and 2008 [24,25].

We found a lack of significant change in rates of knee arthroscopy over time. Data from Canada and England have shown that the utilisation of knee arthroscopy for the treatment of osteoarthritis decreased between 1993 and 2004, but the overall rates of arthroscopy increased in England, and remained steady in Ontario [17]. Similarly, US data comparing 2006 to 1996 found an increase in the overall rate of knee arthroscopy, but a decrease in the rate for osteoarthritis [22]. The decreasing rate of knee arthroscopy was also noted in a review of case logs for the American Board of Orthopaedic Surgery [26]. Another Australian study found an overall reduction of elective knee arthroscopy procedures in Victorian hospitals from 2000–2009 [27].

The rate of TKA within two years of arthroscopy for patients over 64 years of age is higher than figures reported elsewhere. Raaijmaakers et al. [18] found that 17.4% of patients aged 65 years or older required a TKA within 24 months in one hospital in Belgium. In a Canadian study of patients over the age of 50 years at the time of arthroscopy, 9.2% received a TKA within one year and 18.4% within three years [19]. A similar study in Scotland found that over 15.3% of patients over 60 years of age undergoing a knee arthroscopy proceeded to TKA in two years and those regions with the highest rates of arthroscopy also had the highest rates of conversion to TKA within two years [20].

The rate of conversion to TKA within 24 months observed in the over 64 year age group may indicate a lack of effectiveness of arthroscopy in older patients [13]. The decline in the conversion rate of arthroscopy to TKA over the study period may reflect better patient selection, improvements in adjunctive therapy, or longer waiting times for TKA.

A potential limitation of this study is that it relied on administrative datasets. Administrative datasets may comprise incomplete patient data and coding errors. There is also a potential for selection bias due to missing procedure codes. We were unable to obtain the rates of missing procedure codes from the data custodian. Furthermore, the affected side is not reported in these datasets, thus, it is possible that some patients may have had an arthroscopy followed by TKA on the opposite limb. To address this limitation, we used a convenience sample from two of the institutions, covering the same time period as the main study. Of 42 retrieved cases of knee arthroscopies followed by total knee replacements, two cases involved contralateral knees, the remaining cases were ipsilateral. Therefore our rate of conversion to TKA is likely to be an overestimation of the true rate. The indication for knee arthroscopy may affect the likelihood of conversion to TKA, however the indications for knee arthroscopy were poorly reported, thus we were unable to provide such data.

## Conclusions

This study shows decreasing rates of knee arthroscopy in public hospitals and relatively steady rates in private hospitals in an Australian population across a 9-year period. The rates of conversion to TKA following knee arthroscopy were higher than those reported elsewhere, but were decreasing over time.

## Abbreviations

CHeReL: The Centre for Health Record Linkage; NSW: New South Wales; TKA: Total knee arthroplasty

## Competing interests

The authors have no competing interests (financial or non-financial) to declare.

## Authors’ contributions

IAH conceived of the study, ensuring the study abided by initial objectives at all points and helped draft the final manuscript. NSM applied for ethics approval, performed data analyses and helped draft the manuscript. JMN helped with ethics approval, performed data analyses and helped draft the manuscript. SC performed necessary statistical analyses and helped draft the methodology section. RM helped with ethics approval, performed data analyses and helped draft the manuscript. BBJ helped with data analyses and helped draft the manuscript. All authors read and approved the final manuscript.

## Pre-publication history

The pre-publication history for this paper can be accessed here:

http://www.biomedcentral.com/1471-2474/14/143/prepub
